# TiRobot‐Assisted Percutaneous Cannulated Screw Fixation in the Treatment of Femoral Neck Fractures: A Minimum 2‐Year Follow‐up of 50 Patients

**DOI:** 10.1111/os.12915

**Published:** 2021-01-15

**Authors:** Zong‐dong Zhu, Cheng‐wei Xiao, Bo Tan, Xiao‐ming Tang, Dan Wei, Jia‐bin Yuan, Jiang Hu, Liao Feng

**Affiliations:** ^1^ Department of Orthopaedics Sichuan Provincial People’s Hospital, University of Electronic Science and Technology of China Chengdu China

**Keywords:** Avascular necrosis of femoral head, Cannulated screws, Femoral neck fracture, Nonunion, TiRobot

## Abstract

**Objective:**

To assess the long‐term clinical efficacy of TiRobot‐assisted percutaneous cannulated screw fixation in the treatment of femoral neck fractures.

**Methods:**

This retrospective study included 50 patients with unilateral femoral neck fractures who were treated with TiRobot‐assisted percutaneous cannulated screw fixation from September 2017 to May 2018. After at least 2 years of follow‐up, the results of treatment, including operation duration, frequency of fluoroscopy use, intraoperative bleeding, hospital stay, medical expense, screw placement accuracy, rate of fracture healing and necrosis of the femoral head, and Harris hip scores at the last follow up, were recorded and compared with those of 83 matched patients who underwent conventional manual positioning surgery.

**Results:**

The TiRobot group had longer operation duration (83.3 ± 31.2 min *vs* 44.1 ± 14.8 min) and higher medical expenses (28,407.1 ± 7498.0 yuan *vs* 22,672.3 ± 4130.3 yuan) than the conventional group. The TiRobot group had significantly less intraoperative bleeding (11.3 ± 7.3 mL *vs* 51.6 ± 40.4 mL) and shorter hospital stay (8.6 ± 2.8 days *vs* 11.1 ± 3.41 days) than the conventional group. Screw parallelism (1.32° ± 1.85° *vs* 2.54° ± 2.99° on anteroposterior radiograph; 1.42° ± 2.25° *vs* 3.09° ± 3.63° on lateral radiograph) and distance between screws (58.44 ± 10.52 mm *vs* 39.69 ± 12.17 mm) were significantly improved. No significant difference was found between the two groups in terms of the use of fluoroscopy (40.1 ± 28.5 times *vs* 38.6 ± 21.0 times) and Harris hip scores at the last follow‐up (93.2 ± 10.3 points *vs* 88.4 ± 11.9 points). Two cannulated screws penetrated the femoral head during manual insertion in the conventional group but not in the TiRobot group. The rate of nonunion and necrosis of the femoral head in the TiRobot group was reduced compared with that in the conventional group (0 *vs* 7.2%; 6.0% *vs* 24.1%).

**Conclusion:**

TiRobot‐assisted percutaneous cannulated screw fixation of femoral neck fractures is accurate and minimally invasive and helps in reducing late complications, particularly necrosis of the femoral head and nonunion of fractures.

## Graphical abstract

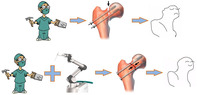

## Introduction

Femoral neck fractures are a commonly encountered injury in orthopaedic practice and result in significant morbidity and mortality. Surgery is the main treatment for femoral neck fractures, as it allows patient mobilization and reduces the risk of complications. For young patients and even for older individuals without serious comorbidities, internal fixation with multiple cannulated screws is the best treatment option because of its advantages such as minimal invasiveness, shorter operative time, and sufficient stability^1^, ^2^, ^3^. Typically, three cannulated screws (6.5, 7.0, or 7.3 mm) are placed in a parallel inverted triangle configuration (inferior, posterosuperior, anterosuperior) with the screws adjacent to the inferior (calcar) and posterior cortices^4^. Parallelism and spread of screws are helpful for fracture compression and stability, thereby promoting healing and reducing the risk of avascular necrosis (AVN) of the femoral head^5^, ^6^. The ideal position of the screws, however, is difficult to achieve by free‐hand empirical screw placement under fluoroscopic monitoring.

Since 1990s, with the development of imageology and computer, robot‐assisted minimally invasive internal fixation has been increasingly applied in orthopaedic surgery^7^, ^8^. Some studies have demonstrated that this technique significantly improves the accuracy of cannulated screw placement in the femoral neck. For example, in the context of a sawbones study in 2012, Müller *et al*. reported that the use of a computer‐assisted planning and navigation system based on three‐dimensional (3D) imaging resulted in a significant reduction in the number of drilling attempts, optimized the accuracy of implant placement by attaining significantly better screw parallelism, and significantly enlarged neck‐width coverage by the three screws^9^.

The “Tianji” orthopaedic surgery robot (TiRobot) was certified by the China Food and Drug Administration in 2016. It has a modular, small, and universal design. This robotic system has provided a breakthrough in 3D perspective navigation in surgery and extends indications for treatment of the spine and orthopaedic traumas. With a positioning accuracy of less than 1 mm, TiRobot can help surgeons complete the placement of cannulated screws efficiently and safely. In 2019, after a mean follow‐up of 13.6 months, Duan *et al*. reported that a TiRobot group had a shorter operation time, less use of fluoroscopy, less intraoperative bleeding, and less total drilling than a conventional surgery group. The screw parallelism was significantly improved and the neck‐width coverage was significantly enlarged compared to the conventional surgery group. However, there was no significant difference between the two groups in terms of the fracture healing rate and Harris scores at the last follow‐up^10^.

Unlike other fractures, there is a higher risk of nonunion and AVN after femoral neck fractures, due to blood supply disruption. Both complications usually occur months to years after surgery and lead to severe deformity. Therefore, reducing these risks is of great significance and a challenge for surgeons. However, whether TiRobot‐assisted percutaneous cannulated screw fixation could reduce these late complications is less clear.

Since September 2017, following the introduction of the TiRobot system, we have been performing robot‐assisted cannulated screw fixation of femoral neck fractures. In this study, we aimed to: (i) analyze the safety and accuracy of the technique; (ii) assess the long‐term clinical efficacy of TiRobot‐assisted femoral neck surgery compared with traditional surgery with a follow up of at least 2 years; and (iii) discuss the surgical precautions, limitations, and further development of robot‐assisted femoral neck surgery.

## Materials and Methods

### *Inclusion and Exclusion Criteria*

The study was approved by our institutional review board. Data of patients who underwent TiRobot‐assisted percutaneous cannulated screw fixation from September 2017 to May 2018 were reviewed (TiRobot group). Moreover, the data was compared with those of patients who had undergone conventional surgery with manual positioning from January 2015 to May 2018 (conventional group). The inclusion criterion was any patient: (i) with closed femoral neck fracture, (ii) treated by internal fixation with three parallel cannulated cancellous screws in our institute; and (iii) who had completed a follow up of at least 2 years. Exclusion criteria were patients who: (i) were without the required complete data; or (ii) refused to participate in this study.

### *Surgical Equipment and Procedure (TiRobot‐Assisted)*

**Fig. 1:**
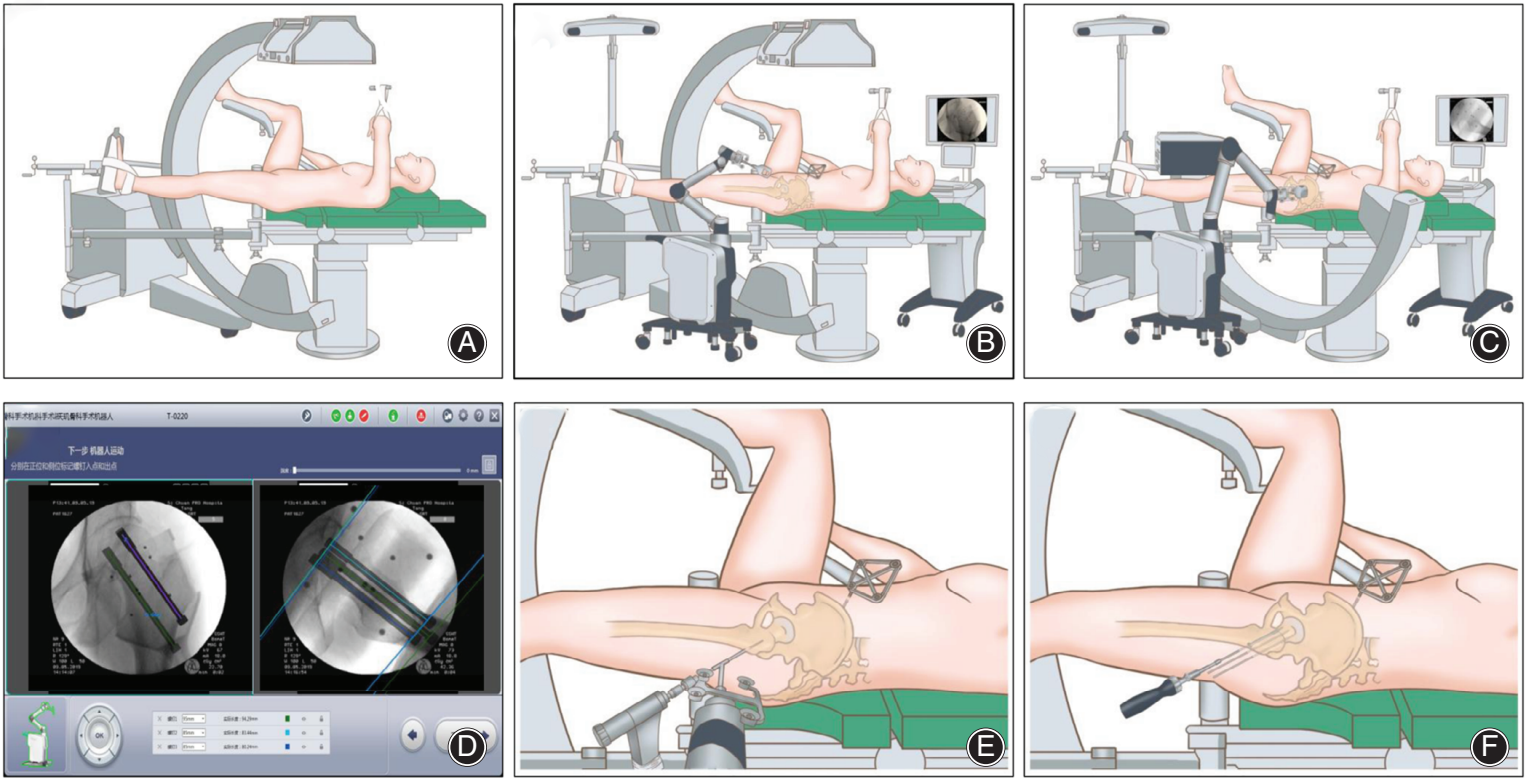
Surgical procedure of TiRobot‐assisted percutaneous cannulated screw fixation of femoral neck fractures. (A) The patient was placed in the supine position on the orthopaedic traction bed, with the injured limb fixed with continuous traction to reduce the fracture. (B, C) The tracker was installed on the robotic arm and placed near the femoral neck. Then, preoperative anteroposterior and lateral fluoroscopy images were obtained and transferred to the system. (D) The surgeon marked the locations of the screws in the software. (E) The system automatically controlled the robotic arm's movement to the planned point. Then, a guiding needle was inserted into the femoral neck through the drill sleeve. (F) Three cannulated screws were inserted in the femoral neck through each guiding needle.

We used the TiRobot system (TINAVI Medical Technologies, China), the C‐arm X‐ray system (Siemens, Germany), and 7.3‐mm‐diameter cannulated screws (Smith & Nephew, UK), strictly following the procedure reported by Wu *et al*.^11^ The surgery procedure is shown in Fig. 1. First, under general anesthesia, the patient was placed in the supine position on the orthopaedic traction bed, with the injured limb fixed with continuous traction. Anteroposterior (AP) and lateral C‐arm X‐ray fluoroscopy was performed to examine the effects of reduction. A tracker was fixed on the ipsilateral anterior superior iliac spine. Second, another tracker was installed on the robotic arm and placed near the femoral neck. All 10 locating points of the tracker were required to be clear and distinguishable in the AP and lateral images. Then, the images were transferred to the system. Third, the surgeon marked the locations of the three screws in the software, which automatically calculated the screw length as a reference. Fourth, the system automatically controlled the robotic arm's movement to the planned point. The drill sleeve was installed and placed into the positioning slot, and a guiding needle was inserted into the femoral neck through the drill sleeve. Finally, the position of the needles was confirmed on X‐rays, and three cannulated screws were inserted through each guiding needle in the following order: below screw, front screw, and rear screw.

### *Surgical Procedure (Conventional)*

After administering the general anesthesia, the patient lay supine on the traction bed, and the fracture was reduced by traction and adduction of the lower limbs. The guiding needles were repeatedly adjusted for the insertion location and angle and were gradually advanced into the femoral neck under fluoroscopic monitoring, until the subchondral bone of the femoral head was reached. The guiding needles were placed cranially in the femoral neck with their distribution presented at an “inverted triangle” layout. After measuring the length, three 7.3‐mm‐diameter cannulated screws were then inserted over the guiding needles.

### *Follow up and Data Collection*

The postoperative regimens were similar between groups. Standard anteroposterior (AP) and lateral hip radiographs were obtained within 24 h after surgery and monthly thereafter. If necessary, CT or MRI were also performed. Walking aids should be used for 8–12 weeks to avoid weight‐bearing movements, until the fracture line is obviously blurred. After at least 2 years of follow‐up, the results of the treatment, including operation duration, frequency of fluoroscopy use, intraoperative bleeding, hospital stay, medical expense, screw placement accuracy, rate of fracture healing and AVN, and Harris hip scores at the last follow up, were recorded and compared with matched patients who underwent conventional manual positioning surgery.

### *Garden Classification*

The Garden classification is based on AP radiographs of the hip and is the most commonly used system for classifying femoral neck fractures. Four types of fractures are categorized as follows: incomplete and valgus impacted (type I), complete and nondisplaced (type II), complete and partially displaced (type III), and complete and fully displaced (type IV)^12^.

### *Pauwels Classification*

The Pauwels classification was the first biomechanical classification for femoral neck fractures. This classification calculates the angle between the fracture line of the distal fragment and the horizontal line to determine the shearing stress and compressive force. Fractures are classified as follows: type I, up to 30°; type II, 30°–50°; and type III, 50° and more^13^.

### *Screw Accuracy*

Based on the acquired images, screw accuracy, including screw parallelism and distance between screws, was evaluated according to the model of Zhu *et al*.^14^. Briefly, a line was drawn running through the core of each screw, and then the angle between every two lines was measured; the sum of the angle was defined as screw parallelism. Similarly, the distance between the screws was defined as the total length of the connecting lines between three screw centers.

### *Nonunion of Fracture*

Fracture healing, which generally requires 4–8 months after reduction and fixation, was judged by two experienced orthopaedic physicians. If no healing was observed after this time, delayed union was considered. If no healing was observed after >9 months and there was no radiographic finding indicating healing for another 3 months, nonunion was considered^15^.

### *Avascular Necrosis of the Femoral Head*

Avascular necrosis of the femoral head was defined as subchondral osteolysis or sclerosis on radiographs or signal changes on MRI, with the presence of persistent/worsening hip pain.

### *Harris Hip Score*

The Harris hip score system was used to assess the postoperative recovery of hip function. The score system mainly includes four aspects: pain, function, absence of deformity, and range of motion. The maximum score is 100 points (best possible outcome). Total scores of <70, 70–80, 80–90, and 90–100 indicate poor, fair, good, and excellent outcomes, respectively^16^.

### *Statistical Analysis*

Quantitative data were expressed as mean ± standard deviation (SD) and were compared using the *t*‐test. Categorical variables were compared by Pearson χ^2^‐test or Fisher's exact test. All analyses were performed with IBM SPSS Statistics for Windows, version 19.0 (IBM, Armonk, NY, USA). *P* ≤ 0.05 was considered significant.

## Results

### *Patient Characteristics*

A total of 50 patients (26 men and 24 women; age range, 12–64 years) were treated with TiRobot‐assisted percutaneous cannulated screw fixation. Of these patients, 3 had diabetes, 1 had renal failure, 1 had nephrotic syndrome, and another 3 had severe osteoporosis. The mean time from injury to operation was 5.4 (range, 1–20) days.

Moreover, 83 had undergone conventional surgery with manual positioning (47 men and 36 women; age range, 17–84 years), and the mean time from injury to operation was 6.0 (range, 1–24) days. Among them, 4 patients had ipsilateral femoral shaft fractures (3 were treated by open reduction and internal fixation with a dynamic compression plate and the other was treated with inverted intramedullary nails), 3 had well‐controlled diabetes, 3 had renal failure (treated with regular dialysis), and 3 had severe osteoporosis.

Patient characteristics are presented in Table 1. No statistically significant difference was found in the general characteristics and fracture classification between the two groups (all *P* > 0.05).

**TABLE 1:** General characteristics of the two groups

Patient characteristics	TiRobot group (50 cases)	Conventional group (83 cases)	*P*
Age (years, mean ± SD)	47.9 ± 13.5	47.7 ± 12.6	0.926
Sex (cases)			0.603
Female	24	36	
Male	26	47	
Garden classification (cases)			0.955
I	11	16	
II	7	10	
III	19	33	
IV	13	24	
Pauwels classification (cases)			0.606
I	5	12	
II	15	28	
III	30	43	
Time from injury to operation (days, mean ± SD)	5.4 ± 3.8	6.0 ± 4.0	0.384

SD, standard deviation.

### *General Results*

The operation duration (starting from reduction to closing the skin) of the TiRobot group was longer than that of the conventional group (83.3 ± 31.2 min *vs* 44.1 ± 14.8 min). Fluoroscopy was used 22–40 (mean, 40.1) times in the TiRobot group, but no significant difference was found when compared with the conventional group, with an average of 38.6 times. Intraoperative bleeding in the TiRobot group ranged from 10 to 50 mL, with an average of 11.3 mL, which was less than that in the conventional group (10–200 mL; mean, 51.6 mL). Hospital stay and medical expenses for patients without comorbidities were 8.6 days and 28,407.1 yuan, respectively, in the TiRobot group, which were also different from those in the conventional group.

### *Screw Accuracy*

For the screw implant placement accuracy, screw parallelism in the TiRobot group was improved (1.32° ± 1.85° *vs* 2.54° ± 2.99° on AP radiograph; 1.42° ± 2.25° *vs* 3.09° ± 3.63° on lateral radiograph) and the distance between screws was increased (58.44 ± 10.52 mm *vs* 39.69 ± 12.17 mm) compared with that in the conventional group. In the conventional group, two cannulated screws penetrated the femoral neck from the inferior side, which was not discovered during surgery, while no similar situation occurred in the TiRobot group. No complications such as wound infection, vascular or nerve injury, screw loosening, or secondary screw displacement were recorded in either group.

### *Clinical and Functional Outcomes*

In the TiRobot group, 5 cases had delayed healing, of which 3 finally healed approximately 12 months after surgery and the other 2 developed AVN of the femoral head at 6 and 12 months after surgery. One patient had necrosis 12 months after surgery when the fracture has healed completely. The aforementioned 3 patients with necrosis had not undergone hip arthroplasty until the final follow up. No case of nonunion was reported in the TiRobot group.

**Fig. 2:**
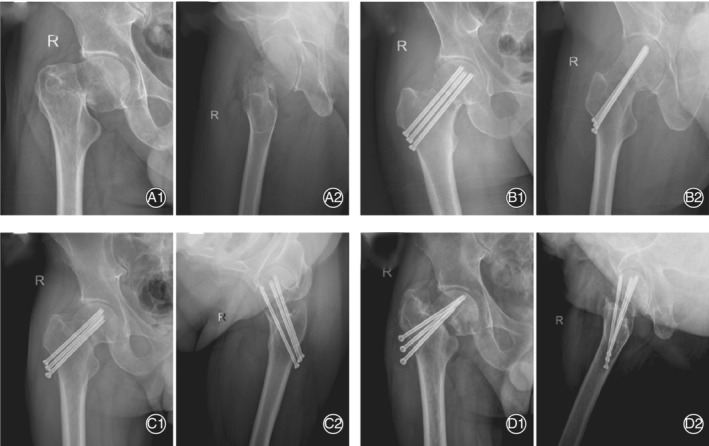
A 52‐year‐old man had nonunion of femur fracture, after internal fixation with three cannulated screws by traditional procedure. (A) Preoperative anteroposterior (AP) and lateral hip X‐ray images; (B) postoperative AP and lateral hip X‐ray images, which show that the cannulated screws are parallel but not spread. (C) Three months after the operation, the fracture had not healed and the screw began slipping. (D) 12 months after the operation, the bone of the femoral neck resorbed and the femoral head was displaced again.

**Fig. 3:**
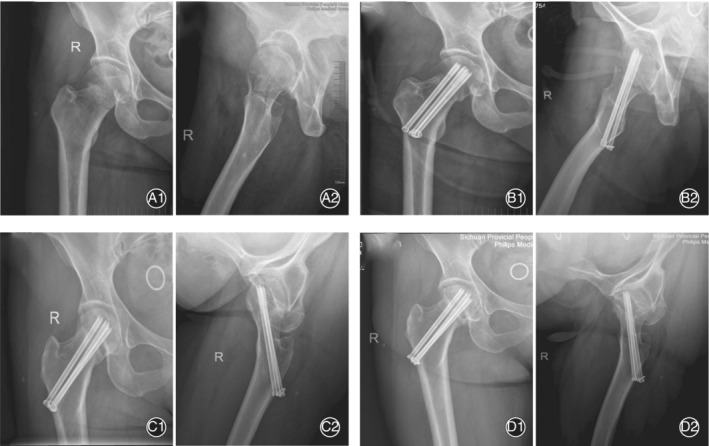
A 53‐year‐old man who had necrosis of the femoral head, after internal fixation with three cannulated screws using the traditional procedure. (A) Preoperative anteroposterior (AP) and lateral hip X‐ray images. (B) Postoperative X‐ray images. (C) 18 months after the operation, the fracture healed. (D) 28 months after the operation, necrosis and collapse of the femoral head could be seen on the radiographs.

**Fig. 4:**
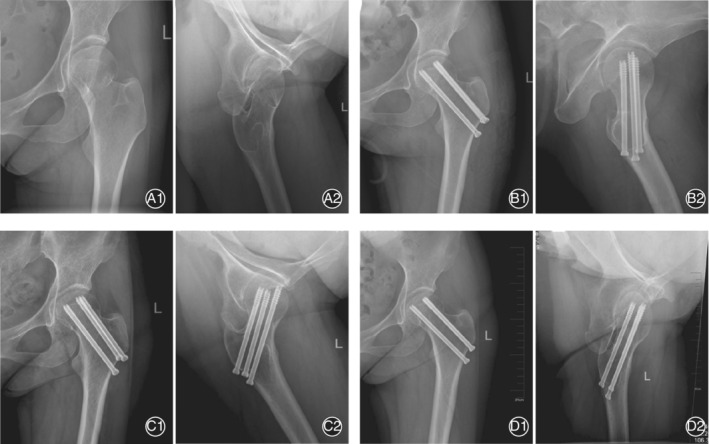
A 46‐year‐old woman, showing that the femoral neck fracture healed smoothly after TiRobot‐assisted percutaneous cannulated screw fixation. (A) Preoperative anteroposterior (AP) and lateral hip X‐ray images. (B) Postoperative X‐ray images, which show that the cannulated screws are parallel and spread. (C) 6 months after the operation, the fracture was healing. (D) 24 months after the operation, the fracture was completely healed without the necrosis of the femoral head.

In the conventional group, 6 patients had nonunion; of these, 1 patient underwent hip arthroplasty 12 months after operation before femoral head necrosis developed. Another 5 patients eventually had avascular necrosis of the femoral head, and all underwent hip arthroplasty. In the conventional group, 10 patients had delayed union, of which 7 finally healed without necrosis and 3 had AVN eventually. Twelve patients had AVN after the fracture healed. In 20 patients, the AVN occurred at 12–29 months, with an average of 20.2 months and a median of 20.5 months. Of the 20 patients with necrosis in the conventional group, 9 underwent hip arthroplasty. Typical cases are shown in Figs 2, 3, 4.

The fracture healing rate in the TiRobot group was higher than that of the conventional group (100% *vs* 92.8%), and the rate of avascular necrosis of the femoral head was significantly low (6% *vs* 24.1%). Some patients complained of hip discomfort when lying on the affected side, but no significant difference was found between the two groups in Harris hip scores at the last follow up. A comparison of results is shown in Table 2.

**TABLE 2:** Comparison of results in the two groups

Results	TiRobot group (50 cases)	Conventional group (83 cases)	*P*
Operation duration (min, mean ± SD)	83.3 ± 31.2	44.1 ± 14.8	0.000
Fluoroscopy frequency (times, mean ± SD)	40.1 ± 28.5	38.6 ± 21.0	0.873
Intraoperative bleeding (mL, mean ± SD)	11.3 ± 7.3	51.6 ± 40.4	0.000
Hospital stay for patients without comorbidities (days, mean ± SD)	8.6 ± 2.8	11.1 ± 3.4	0.000
Medical expenses for patients without comorbidities (yuan, mean ± SD)	28407.1 ± 7498.0	22672.3 ± 4130.3	0.000
Screw parallelism (AP, degree)	1.32 ± 1.85	2.54 ± 2.99	0.005
Screw parallelism (lateral, degree)	1.42 ± 2.25	3.09 ± 3.63	0.001
Distance between screws (mm)	58.44 ± 10.52	39.69 ± 12.17	0.000
Follow‐up time (months, mean ± SD)	33.7 ± 6.0	41.8 ± 11.4	0.000
Nonunion (cases)	0	6	0.083
Necrosis of femoral head (cases)	3	20	0.008
Harris score (points, mean ± SD)	93.2 ± 10.3	88.4 ± 11.9	0.176

AP, anteroposterior; SD, standard deviation.

**Fig. 5:**
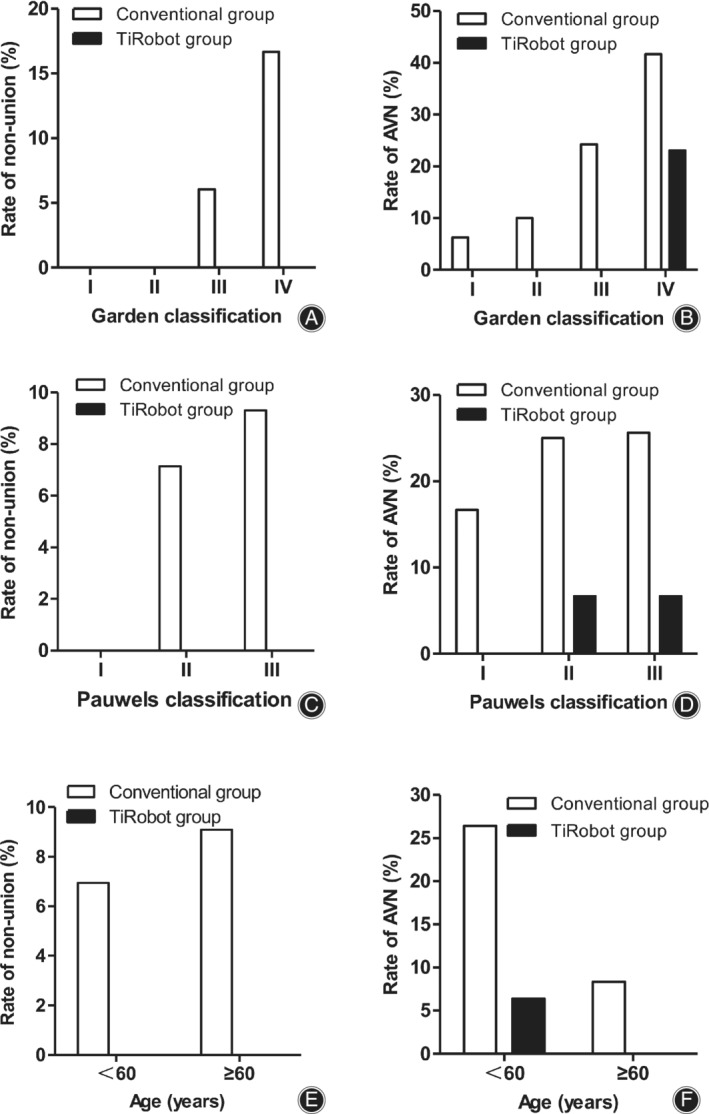
Subgroup analyses. (A) Nonunion rates for fractures stratified by the Garden classification. (B) Avascular necrosis (AVN) rates for fractures stratified by the Garden classification. (C) Nonunion rates for fractures stratified by the Pauwels classification. (D) AVN rates for fractures stratified by the Pauwels classification. (E) Nonunion rates for different age groups. (D) AVN rates for different age groups.

The main factors affecting the prognosis of femoral neck fractures are the fracture subtype and the patient's age. Therefore, we further analyzed the results according to age and fracture subtype. As shown in Fig. 5, nonunion and AVN was rare among patients with Garden type I or II fractures and common among patients with Garden type III or IV fractures. No case of nonunion was observed in the TiRobot group, and the AVN rate for patients with type III or IV fractures was lower than that for the conventional group. Subanalysis according to the Pauwels type yielded similar results. Further analysis of patients by age showed that TiRobot‐assisted surgery can reduce the rate of nonunion and AVN in all age groups.

## Discussion

With the development and advances in medical imaging and computer technologies, computer‐assisted orthopaedic surgery has been used worldwide^8^. The stereotactic technique based on radiograph or 3D CT images can assist doctors to perform more accurate surgical planning, avoid errors of manual operation, and reduce radiation exposure^9^, ^17^, ^18^, ^19^. Recently, with the combination of the stereotactic technique and automatic manipulator, versatile state‐of‐the‐art robot‐based navigation systems for orthopaedic surgery have been developed, such as the “TianJi” robotic system. Given the advantages of being simple, precise positioning, minimal invasiveness, and minimal radiation exposure, robot‐assisted orthopaedic surgery is being accepted by an increasing number of doctors. A few studies have reported its advantages in traumatic orthopaedics^10^, ^20^, ^21^, ^22^, ^23^.

Consistent with previous studies, we found that those in the TiRobot group had less intraoperative bleeding and shorter hospital stay^10^. However, if anatomical reduction cannot be achieved by closed reduction, more frequent bleeding is still inevitable during open reduction. Unlike previous reports, we found that the frequency of using intraoperative fluoroscopy in the TiRobot group did not decrease, while the operation time increased significantly. This is because robotic surgery requires additional surgical steps, and in the early period in which the system was first introduced, the surgeons were not skillful enough. The frequency of using intraoperative fluoroscopy is mainly related to the repeated confirmation required when inserting the guiding needle and cannulated screws into the femoral head, while the TiRobot system cannot assist in controlling the depth of insertion. Therefore, no difference was found in the above data between the two groups.

A previous study reported that parallelism and spreading of cannulated screws are important for fracture compression and stability and help in fracture healing^24^. However, the ideal position of the screws is difficult to achieve by free hand under fluoroscopic monitoring. The invention of the TiRobot completely solved this problem; therefore, late complications of fractures are significantly reduced. Parallel screws allow femoral neck compression and collapse as the fracture heals, so some patients complained of hip discomfort when lying on the affected side, which was caused by screw slipping and can be solved by removing the screws after fracture healing.

Consistent with the findings in previous studies, our findings revealed lower nonunion and AVN rates for patients with Garden type I or II (stable type) fractures than for those with type III or IV (unstable type) fractures. However, unlike other studies, the AVN rate for old patients in this study was lower than that for young patients. This is because all elderly patients included in this study had stable fractures, while some of the young patients had unstable fractures, which led to bias. Nevertheless, this study demonstrated that TiRobot‐assisted surgery can reduce the risk of nonunion and AVN in all patients.

At present, the main function of the “TianJi” robotic system is limited to indicating the designed slot by drill sleeve on the manipulator automatically, so the surgeon can insert the guiding needle into the bone through the drill sleeve. With the continuous development and functional improvement of orthopaedic robot systems, more functions may be realized, such as planning the screw position automatically, controlling the depth when drilling the needle, and even completing some steps of operations automatically^7^.

### *Surgical Experiences*

The surgical experiences of our institute may be summarized as follows. First, the quality of reduction is an important factor in determining fracture healing and AVN of the femoral head^25^, but the robot cannot assist in reduction; therefore, the first step of surgery should be anatomical reduction. Second, for obese or muscular patients, the subcutaneous tissue and fascia should be bluntly separated, before drilling the guiding needle, to avoid bias. Third, because the guiding needle is at an obtuse angle to the cortical bone, it will slide proximally when drilling, so the planned position of the screw needs to be slightly inferior and drilling should be done gently.

### *Study Limitation*

Avascular necrosis of the femoral head usually appears 2–3 years after femoral neck fracture^26^. Of the 23 patients with necrosis in this study, 22 had positive clinical and imaging evidence of necrosis within 2 years and 1 patient was diagnosed 29 months after surgery by radiography, but hip pain had appeared months before necrosis was found. Therefore, the risk of necrosis is low in asymptomatic patients after at least 2 year of follow up. Although the follow‐up time of the TiRobot group was shorter than that of the conventional group, it has little effect on the results.

This study only presented results from a single‐center experience, as there is a lack of multicenter‐controlled studies. In the two groups, surgery was not performed in the same period, and the surgeon's experience, technology, and medical costs changed. Further prospective multicenter randomized controlled studies with large numbers of patients are needed.

## Conclusion

The TiRobot system improves the accuracy of cannulated screw placement. TiRobot‐assisted percutaneous cannulated screw fixation of femoral neck fractures helps in reducing late complications, particularly necrosis of the femoral head and nonunion.
